# Advanced Diagnostic Tools in Hypothermia-Related Fatalities—A Pathological Perspective

**DOI:** 10.3390/diagnostics14070739

**Published:** 2024-03-30

**Authors:** Andreea Alexandra Hleșcu, Adriana Grigoraș, Victor Ianole, Cornelia Amalinei

**Affiliations:** 1 Legal Medicine Department, Faculty of Medicine, “Grigore T. Popa” University of Medicine and Pharmacy, 700115 Iasi, Romania; andreea.velnic@yahoo.com; 2 Department of Morphofunctional Sciences I, “Grigore T. Popa” University of Medicine and Pharmacy, 700115 Iasi, Romania; ianole.victor@gmail.com; 3 Department of Histopathology, Institute of Legal Medicine, 700455 Iasi, Romania

**Keywords:** hypothermia, S100β, Hsp70, ICAM-1, AQP1, forensic pathology, autopsy

## Abstract

Background and Objectives: Although classical gross features are known in hypothermia victims, they lack specific diagnosis features. The aim of our study was to reveal specific brain and lung pathological features in a group of hypothermia-related fatalities. Materials and Methods: The study group comprised 107 cases from our files associated with hypothermia. Routine hematoxylin–eosin (H&E) staining and postmortem immunohistochemistry were performed. Results: The microscopic cerebral exam revealed diffuse perineuronal and perivascular edema, gliosis, mononuclear cell infiltration, acute brain injuries, focal neuronal ischemia, lacunar infarction, and variable hemorrhages. Variable alveolar edema, pulmonary emphysema, intra-alveolar and/or pleural hemorrhage, and bronchopneumonia, as well as other pre-existing lesions, were identified in lung tissue samples. Glial cells displayed S100β expression, while neurons showed moderate Hsp70 immunopositivity. Alveolar basal membranes exhibited diffuse ICAM-1 positive expression, while ICAM-1 and AQP-1 positivity was observed in the alveolar septum vascular endothelium. Statistical analysis revealed a significant correlation between S100β and Hps70 immunoexpression and cerebral pathological features, between ICAM-1 immunoexpression and alveolar edema and pulmonary emphysema, and between AQP-1 immunoexpression and pulmonary emphysema. Conclusions: Our results add supplementary data to brain and lung pathological findings in hypothermia-related fatalities, with potential therapeutic value in hypothermia patients.

## 1. Introduction

Hypothermia is characterized by a decreased body temperature, below 35 °C, due to the imbalance between the production and loss of body heat [1]. Thermal stress has variable features in forensics practice, representing a domain of interest for research, considering its complex interrelationships with pathological diagnosis and causes of death. The body’s exposure to low environmental temperatures represents a stress factor, which increases the metabolic rate and leads to the excessive production of reactive oxygen species (ROS) [2]. ROS induce oxidative stress, as a consequence of overcoming cellular antioxidative defense mechanisms [2]. Additionally, thermal stress is associated with tissue hypoxia and the disruption of the physiological activity of vital organs, such as the brain, lungs, heart, and kidneys [1,3,4]. The decreased body temperature below 30 °C is accompanied by an insidious onset of some neurological manifestations, such as lethargy, hampered speech, loss of coordination, ataxia, confusion, and unconsciousness, manifestations that may progress toward coma and death in severe hypothermia cases [5,6]. These neurological manifestations are basically the expression of nonspecific cerebral lesions, such as perineuronal edema, intraparenchymal congestion, and perivascular hemorrhages, along with endothelial, neuronal, and glial changes [7,8].

The glial cells’ reactivity is pivotal in the neuroprotective response to acute brain injuries that occur in accidental hypothermia stress or therapeutic hypothermia [7,9]. S100 calcium-binding protein B (S100β) is a cytoplasmic calcium-binding brain protein, mainly produced by astrocytes [7,10,11]. Its role in cerebral protection and postlesional repair has recently been recognized, being recommended as one of the most specific astrocyte markers according to investigations of its association with depression, brain trauma, thermal stress, and neurodegenerative diseases, e.g., Alzheimer’s disease [7,10,11]. Ischemia and hypothermic stress may lead to a heat-shock-like response [12]. As a central component of chaperons’ intracellular network, heat shock protein 70 (Hsp70) is a neuroprotective factor involved in assisting protein folding, translocation, and degradation [13,14]. Postmortem studies have revealed the induction of Hsp70 expression in the human brain when exposed to low-temperature conditions, representing an indicator of cellular stress [12,15].

Hypothermia or other instances of alveolar hypoxia initially lead to interstitial edema, followed later on by alveolar edema [16]. Pulmonary edema, as an expression of pulmonary and systemic hemodynamic perturbations, associated with microvascular bed lesions, indicates the progression toward multiple organs’ dysfunction and, finally, death [17]. Intercellular adhesion molecule 1 (ICAM-1) or CD54 is an adhesion molecule expressed both in the alveolar epithelium and the alveolar endothelium, which plays an important role in the local recruitment of neutrophils and their lung trafficking [17]. Additionally, Aquaporin 1 (AQP1) intervenes in alveolar endothelium permeability control, its positivity in the lung parenchyma suggesting AQP1 involvement in edema onset in patients with hypothermia [17].

Despite the efforts to decipher the complex molecular mechanisms related to cerebral and pulmonary lesion development and their morphological features related to cold stress, they remain partially unknown. In this context, the present study is focused on the cerebral and pulmonary lesions’ evaluation, along with the postmortem immunohistochemical analysis of S100β, Hsp70, ICAM-1, and AQP1 expression in a large group of hypothermia-related victims in order to explore their potential role in hypothermia diagnosis in forensic medicine.

## 2. Materials and Methods

This research was performed by complying with the Declaration of Helsinki—Ethical Principles for Medical Research Involving Human Subjects, with the approval of “Grigore T. Popa” University of Medicine and Pharmacy, Iasi, Research Ethics Committee no. 17275/20 obtained in August 2019.

**Figure 1:**
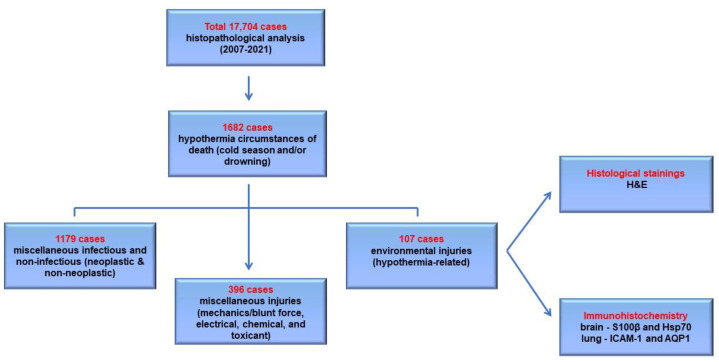
The selection of cases and methods applied for hypothermia-related victims. AQP1—aquaporin 1; H&E—hematoxylin–eosin; Hsp70—heat shock protein 70; ICAM-1—intercellular adhesion molecule 1; S100β—S100 calcium-binding protein B.

The review of the reports of our department led to the selection of 17,704 cases that required histopathological analysis, for a period of 15 years, from 2007 to 2021. Our search identified 1682 cases of hypothermia circumstances of death (cold season and/or drowning). Among these, 1179 of these cases were diagnosed with miscellaneous death-related pathologies, while 396 of them had death-related miscellaneous injuries (Figure 1).

After the exclusion of these cases, 107 cases of profound hypothermia victims were included in our group, in the appropriate circumstances of death. The autopsy exam was associated with cerebral samples collected from nontraumatic and grossly nonlesional brain cortex areas in 107 cases and lung tissue samples collected from only 106 cases, due to hemicorporectomy registered in a victim. The lung tissue samples were taken from both grossly lesional and nonlesional subpleural and perihilar areas.

The collected specimens were investigated by hematoxylin–eosin (H&E) routine staining and postmortem immunohistochemistry for S100β [(monoclonal antibody, mouse, 9A11B9/Thermo Fisher Scientific, Shanghai, China), dilution 1/600, with nuclear and cytoplasmic staining pattern], and Hsp70 [(monoclonal antibody, rabbit, T.527.6/Thermo Fisher Scientific, San Diego, CA, USA), dilution 1/400, with cytoplasmic staining pattern] for brain tissue samples, added with ICAM-1 [(monoclonal antibody, human, 15.2/Thermo Fisher Scientific, USA), dilution 1/50, with membrane staining pattern], and AQP1 [(monoclonal antibody, human, OTI2D10/Thermo Fisher Scientific, China), dilution 1/300, with membrane staining pattern] for pulmonary tissue samples. Fresh 4 μm sections of each formalin-fixed, paraffin-embedded sample were deparaffinized in xylene, followed by rehydration by immersion in four successive alcohol baths, from 100% to 70% concentrations, for postmortem immunohistochemistry. The heat antigen retrieval was achieved with 10 mM sodium citrate buffer (pH = 6). Incubation with UltraVision Hydrogen Peroxide Block (Thermo Scientific, UltraVision LP Kit, TL-06-HL, The Netherlands) was performed for 10 min to reduce endogenous peroxidase, followed by buffer wash and UltraVision Protein Block for 5 min, to block nonspecific background staining. The slides were incubated overnight at 4 °C, following each primary antibody application. The application of Primary Antibody Amplifier Quanto (Thermo Scientific, UltraVision Quanto Detection System HR DAB, The Netherlands) was performed for 10 min, followed by treatment with horseradish peroxidase (HRP) Polymer Quanto for 10 min, with buffer washes between incubations. The immune reaction was developed with 3,3′-Diaminobenzidine (DAB) Quanto Chromogen solution in DAB Quanto Substrate for 5 min, followed by Mayer’s hematoxylin counterstaining. The negative control was performed by the omission of the primary antibody. Normal and tumor tissue samples were used for internal positive control, according to the manufacturer’s recommendations. The postmortem immunohistochemical results were interpreted by two independent pathologists for each slide, as an average percentage in three fields under 200× magnification, according to the available literature data [7], the reaction being considered as positive when ≥25% of cells showed positive staining.

### Statistical Analysis

The specific variables of statistical analysis were calculated using IBM SPSS Statistics 26 version software. Chi-squared and Fisher’s exact test, at the critical significance level *p* < 0.05, were used to test the correlations between specific parameters.

## 3. Results

The study group comprised 107 cases of profound hypothermia-related victims, 24% (*n* = 26) women and 76% (*n* = 81) men, with a mean age of 57.95 ± 1.47 years. Overall, 30.84% (*n* = 33) of hypothermia-related deaths occurred in patients who were 61–70 years old, followed by 23.36% (*n* = 25) in patients who were 51–60 years old, 13.08% (*n* = 14) in patients who were 71–80 years old, and 11.21% (*n* = 12) in patients who were 41–50 years old. Notably, 14.95% (*n* = 16) of victims were ≤ 40 years old. Most of them were between 31 and 40 years old (10.28%; *n* = 11), and 4.67% (*n* = 5) were 21-30 years old, while the youngest case was represented by a 10-year-old victim (0.93%), in our study group.

Other data related to the risk factors, different pre-existing diseases, and demographic features, along with biochemical and toxicological results, have been previously reported [3].

### 3.1. Cerebral Pathological and Postmortem Immunohistochemical Features

The gross autopsy findings in brain examination revealed cerebral congestion, edema, subarachnoid hemorrhage, cerebral hemorrhages, and brain softening, along with scalp hematomas, in addition to pre-existing cerebral atherosclerosis, as illustrated in Table 1.

**Figure 2:**
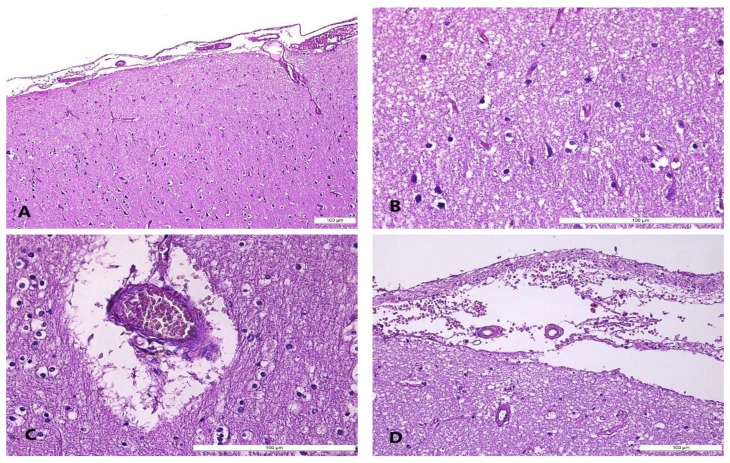
Cerebral microscopic features in hypothermia cases: (**A**) perineuronal and perivascular edema; (**B**) perineuronal edema and focal neuronal ischemia; (**C**) congestion and perivascular edema; (**D**) subarachnoid hemorrhage and perineuronal edema in the cerebral cortex; hematoxylin–eosin (H&E) staining.

The microscopic features certified the gross features, adding more microscopic-sized lesions, with other findings represented by arteriolosclerosis and meningeal fibrosis as pre-existing pathologies. In addition, gliosis and mononuclear cell infiltration, acute brain injuries, and focal neural ischemia, along with intraparenchymal and perivascular hemorrhages, were also registered (Figure 2 and Table 2).

**Figure 3:**
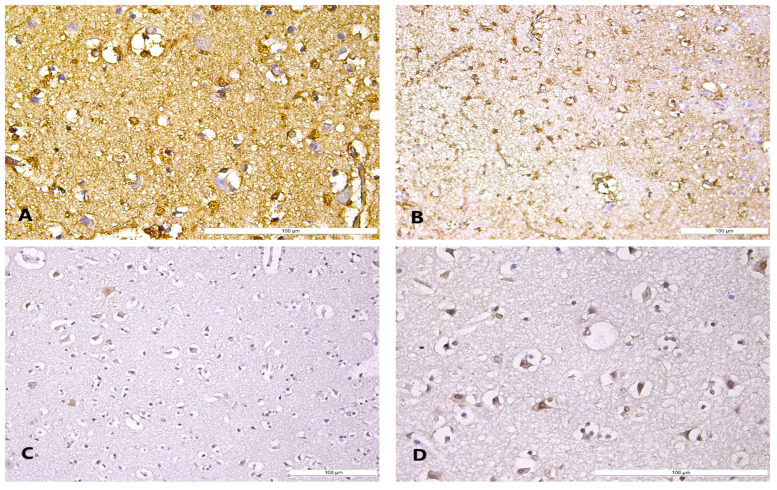
Cerebral S100β and Hsp70 expression in hypothermia cases: (**A**) cytoplasmic and focal nuclear S100β expression in glial cells; (**B**) S100β expression glial cell positivity predominantly identified in the cerebral cortex; (**C**) moderate Hsp70 neuronal positivity in the cerebral cortex; (**D**) moderate neuronal Hsp70 cytoplasmic positivity compared to associated glial cell negativity in the cerebral cortex.

The postmortem immunohistochemical examination revealed S100β glial cell positivity, with the heterogeneity of immunohistochemistry patterns showing cytoplasmic and focal nuclear expression, predominantly disposed in the cerebral cortex compared to the white matter expression (Figure 3). Additionally, the neurons displayed a negative S100β immunoexpression. In an analogous pattern, the neurons showed a moderate Hsp70 cytoplasmic immunopositive reaction compared to associated glial cells, mainly registered at the margins of cerebral ischemic areas (Figure 3).

The statistical analysis revealed a significant correlation between S100β expression and diffuse perineuronal and perivascular edema (*r* = 0.34, *p* = 0.019), arteriolosclerosis (*r* = −0.80, *p* < 0.001), gliosis and mononuclear cell infiltration (*r* = −0.42, *p* = 0.001), lacunar infarction (*r* = −0.48, *p* < 0.001), subarachnoid hemorrhage (*r* = −0.65, *p*< 0.001), and intraparenchymal and perivascular hemorrhages (*r* = −0.34, *p* = 0.003).

In addition, a significant correlation was observed between Hsp70 expression and arteriolosclerosis (*r* = −0.62, *p* < 0.001), gliosis and mononuclear cell infiltration (*r* = −0.35, *p* = 0.001), acute brain injuries (*r* = −0.27, *p* = 0.020), focal neuronal ischemia (*r* = −0.27, *p* = 0.020), lacunar infarction (*r* = −0.31, *p* = 0.005), subarachnoid hemorrhage (*r* = −0.48, *p* < 0.001), and intraparenchymal and perivascular hemorrhages (*r* = −0.53, *p* < 0.001) (Table 2).

### 3.2. Pulmonary Pathological and Postmortem Immunohistochemical Features

Pulmonary congestion and edema, along with bullous emphysema, lung hemorrhages, pleural petechiae, and purulent inflammation, in addition to chronic parenchymal and pleural inflammation and fibrosis were the gross features identified in our study group (Table 3).

**Figure 4:**
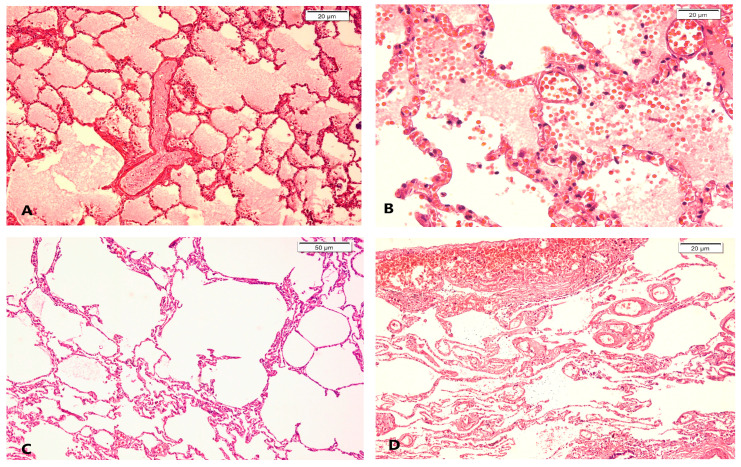
Pulmonary microscopic features in hypothermia cases: (**A**) diffuse alveolar edema; (**B**) capillary congestion and hemorrhagic edema; (**C**) emphysema on a variable autolytic background; (**D**) pleural hemorrhages and congestion; hematoxylin–eosin (H&E) staining.

The most relevant lung microscopic features were hemorrhagic or marked diffuse alveolar edema, areas of pulmonary emphysema, and intra-alveolar and/or pleural hemorrhages, all diagnosed on a variable autolytic background, as illustrated in Figure 4 and Table 4. Areas of pleural adhesions accompanied by chronic fibroinflammatory lesions or pulmonary tuberculosis, as pre-existing lesions, were also noticed. Bronchopneumonia with/without abscess formation was also observed in our group, associated with acute respiratory distress syndrome (ARDS) in a case.

**Figure 5:**
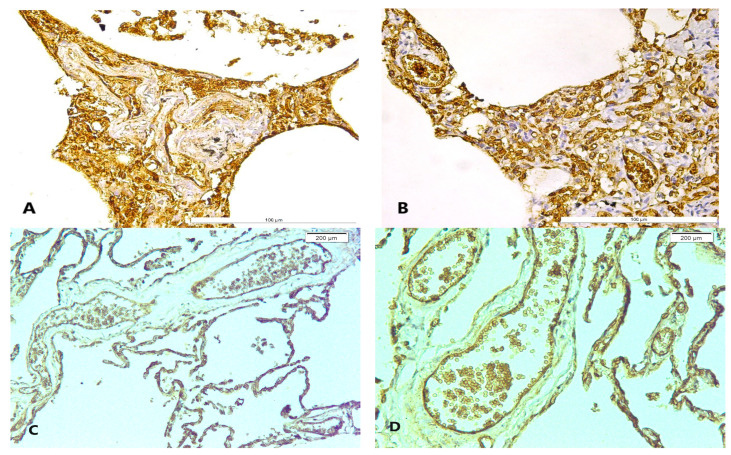
ICAM-1 and AQP-1 pulmonary expression in hypothermia cases: (**A**,**B**) ICAM-1 membrane expression in alveolar walls, predominately disposed in basal alveolar membranes and vascular endothelium; (**C**,**D**) AQP-1 intense positive immunoexpression in vascular endothelial cells of the alveolar septa.

Diffuse ICAM-1 membrane positive expression in alveolar walls, predominately disposed in the basal alveolar membranes and vascular endothelium, was detected in all cases (Figure 5). Additionally, a strong AQP-1 immunostaining was also observed in all vascular endothelial cells in our study group (Figure 5).

A significant statistical correlation was registered between ICAM-1 immunoexpression and hemorrhagic alveolar edema (r = 0.20, *p* = 0.034), marked diffuse alveolar edema (r = −0.20, *p* = 0.034), pulmonary emphysema (r = −0.36, *p* = 0.001), pleuritis (r = −0.55, *p* < 0.001), and pleural adhesions (r = −0.50, *p* < 0.001). Additionally, no statistical correlation was found between AQP1 immunoexpression and lung pathological features, with the exception of pulmonary emphysema (r = −0.27, *p* = 0.045) (Table 4).

## 4. Discussion

The balance between environmental heat gain added to the basal metabolic heat rate versus heat loss results in the normal body temperature of 36.8 ± 0.9 °C [18]. In cases of prolonged exposure of human organisms to subfreezing temperatures (0 °C), or if the wind chill factor is increased, even at above-freezing environment temperatures, hypothermia may occur [3,18]. A spectrum of hypothermia grades, from grade I to IV, are registered, in association with various pathophysiologic lesions. Accordingly, hypothermia grade I (mild) is encountered when the core temperature ranges between 35 and 32 °C, hypothermia grade II (moderate) is registered when the core temperature ranges between 32 and 28 °C, and hypothermia grade III (severe) is diagnosed when the core temperature is lower than 28 °C. The last stage, hypothermia grade IV (profound), is encountered when the core temperature is lower than 24 °C, correlated to a functional poikilothermic state [2,18,19,20]. Considering the profound hypothermia stage registered in our study group, it contributed to the fatal progression due to severe complications, despite the eventual patient hospitalization and therapy [3].

Patients with hypothermia show progressively installed neurological manifestations, which can progress to coma and death [1]. They most often appear when the brain temperature progressively decreases, considering that a 1 °C drop in temperature is associated with a 6-8% reduction in cerebral metabolism, similar to the results obtained in an experimental model [21].

Additionally, exposure to cold stress induces a local cerebral protective response expressed by cerebral blood flow autoregulation to allow for the maintenance of adequate cerebral circulation, even if the body temperature drops to approximately 25 °C [5]. These neurological manifestations are essentially the expression of cerebral tissue, which are also recorded in some cases of death due to severe hypothermia [2,8]. The lesions are considered to be associated with the brain tissue response to a reduced cerebral blood flow due to intravascular sludge and microcirculation vasoconstriction [2]. The reduced cerebral metabolic rates in the context of hypothermia is added to the neurotoxic effects attributed to catecholamine metabolites, free radical excess, and possible to extracellular fluid freezing [2]. Histological features identified in an experimental animal model showed brain tissue architecture alteration and hemorrhages, along with cell damage, such as glial cell damage and pyramidal neuron vacuolar degeneration, correlated to the degree of hypothermia [8]. The cerebral lesions most frequently identified in different forensic studies were perineuronal and perivascular edema and microhemorrhages associated with neuronal ischemia, which may progress to necrosis and inflammation [1,2,8]. These literature data are in agreement with the results of our study, showing perineuronal and perivascular edema and intraparenchymal and perivascular hemorrhages associated with focal neuronal ischemia and acute brain injuries in three cases. These lesions were possibly more extensive in our research, considering victims’ pre-existing cerebral vascular lesions, such as cerebral arteriolosclerosis, which might have resulted in the faster progression of cerebral lesions.

The reactivity of glial cells plays a central role in the neuroprotective response to hypothermic stress, S100β being a marker of astrocyte activation [22]. Although S100β, at low concentrations, displays a neuroprotective effect [22], the release of large amounts of S100β by astrocytes in prolonged cerebral ischemia has a neurotoxic effect, a feature possibly observed in patients with severe hypothermia [22]. Moreover, the literature data regarding the postmortem immunohistochemical S100β expression in cases of hypothermia are limited [7]. In this direction, Wang et al. observed an increased immunoexpression of fibroblast growth factor β (FGF-β) and S100β in the glial cells of the cerebral cortex as a local tissue response to thermal stress [7]. These data support the results of our research, demonstrating S100β glial cell positivity, a finding observed predominantly in the cerebral cortex, while the associated neurons showed a negative immunoreaction. Moreover, the statistical results revealed a strong correlation between S100β immunoexpression and gliosis, along with mononuclear cell infiltration (*p* = 0.001), intraparenchymal and perivascular hemorrhages (*p* = 0.003), and diffuse perineuronal and perivascular edema (*p* = 0.019) in the cerebral cortex, most probably related to the nervous tissue response to ischemia.

The cellular response to heat-induced stress is also manifested by an increased expression of heat shock proteins (Hsps) or chaperones. Among these, Hsp70 and Hsp90 display a neuroprotection effect in ischemic stroke models [12,14]. Although studies focusing on the link between hypothermia and chaperone expression are relatively limited, molecular tests showed that Hsp70 overexpression is associated with increased neuronal resistance and faster regeneration following hypoxia-induced cerebral cortical injuries [23]. Moreover, cerebral cells’ Hsp overexpression was observed in therapeutic hypothermia [24], while a combined treatment of hypothermia with valproic acid significantly upregulated Hsp70 expression compared to that induced only by hypothermia [12]. In the same direction of research, the study carried out by Tirapelli et al. demonstrated a moderate neuronal Hsp70 immunopositivity in rodents exposed to cerebral hypoxia induced by hypothermia and in hypothermia associated with the administration of ketoprofen, compared to a control group [25]. Additionally, endothelial cells of hypoxic cerebral vessels of variable etiologies release extracellular vesicles containing microRNAs (miRs) and Hsp70, which directly protect neurons from ischemia or reperfusion injury by inhibiting apoptosis and stimulating growth and cell migration [26]. Additionally, experimental studies have demonstrated a variable neuronal intensity of Hsp70 expression, while its predominant location was at the margins of cerebral ischemic areas [27,28]. This pattern suggests that it may be an indicator of the cellular stress level, all the more as the deletions of the Hsp70 gene are associated with an enhancement of hypoxic-induced neuronal apoptosis [27,28]. All these data are consistent with the results of our study showing a moderately positive Hps70 reaction, predominantly located in neurons, which demonstrates the adaptative reaction of brain tissue to hypothermic-induced hypoxic conditions. Furthermore, the statistical analysis results demonstrated a strong correlation between Hsp70 immunoexpression and focal neuronal ischemia (*p* = 0.020), acute brain injuries (*p* = 0.020), and intraparenchymal and perivascular hemorrhages (*p* < 0.001) that support the local neuronal adaptation to the hypothermia-related stress.

Hypoventilation is followed by CO_2_ accumulation, which subsequently leads to hypoxia and respiratory acidosis in patients with hypothermia [4]. There is an approximately 50% reduction in O_2_ consumption and CO_2_ production, respectively, with anatomical respiratory dead space being increased through bronchial dilation but with unchanged alveolar dead space if the body temperature drops below 30 °C [29]. However, gas exchange is not affected, despite the increased resistance of the pulmonary vascular bed and the disruption of the alveolar ventilation–perfusion rate in moderate hypothermia [29]. If the body temperature continues to decrease, hypoventilation is followed by apnea, hypoxia, and acidosis, which predispose to severe arrhythmias [4]. Thermally induced alveolar hypoxia leads to the onset of pulmonary edema, initially in the interstitium and, subsequently, in the alveolar space [16]. The onset of pulmonary edema, as an expression of disturbances in pulmonary and systemic hemodynamics, accompanied by microvascular bed lesions, is a strong indicator of the progression toward multiple organ dysfunction and ultimately death [17]. Moreover, pulmonary edema was the main gross and microscopic lesion observed in our studied group. Alveolar edema mostly showed a focal distribution (82.24% of the cases), while its distribution was grossly diffuse in only 16.82% of the cases.

The literature contains relatively scarce data regarding the ensemble of autopsy morphological changes in the interalveolar septum that appear in fatal hypothermia, with these being mainly focused on the therapeutic applicability of exposure to low temperatures (therapeutic hypothermia) in the context of different injuries by reducing alveolar septum leukocyte infiltration and increasing the proportion of dipalmitoylphosphatidylcholine upon compression [30,31].

However, morphological studies aimed to identify the structural lesions in the lung parenchyma exposed for more than 12 h to hypothermic Euro Collins solution highlighted the presence of vacuoles in type I pneumocytes and endothelial cells of the alveolar capillaries, interstitial edema, the loss of the apical microvilli, and pneumocyte type II lamellar degeneration [32,33]. Moreover, endothelial and alveolar epithelial lesions contribute to the disruption of fluid flow from the interalveolar septum and the onset of pulmonary edema. Other structural changes in the alveolar septum, such as local leukocytic infiltration and the activation of the coagulation cascade, as a consequence of endothelial cell damage, also compete with this process [34].

ICAM-1, an intercellular adhesion molecule, a member of the immunoglobulin superfamily, and the main ligand of the lymphocyte function-associated antigen 1LFA-1, expressed by leukocytes, plays an important role in the tissue regulation of leukocyte traffic [35]. It is expressed mainly by the vascular endothelium and type I pneumocytes of the alveolar septum, and its overexpression is induced by a series of proinflammatory cytokines released by alveolar macrophages, such as tumor necrosis factor α (TNF-α) and interleukin 6 (IL-6) [36]. Although some studies carried out on a murine model revealed that leukocyte infiltration of the alveolar septum and ICAM-1 expression is reduced via the activation of ERK1/2 and the inhibition of the STAT3 pathway in mild hypothermia [35,37], ICAM-1 overexpression contributes to the development of interstitial edema by neutrophil recruitment and trafficking in the alveolar septum in severe hypothermia [17]. The neutrophil–epithelial cell interaction can lead to the tyrosine phosphorylation of tight junction proteins, which results in alveolar epithelium and capillary endothelium permeability increase [34]. The results of our study demonstrated the diffuse ICAM-1 overexpression in the alveolar septum, in agreement with the literature data [17]. In addition, the statistical analysis revealed a significant correlation between ICAM-1 immunoexpression and hemorrhagic alveolar edema (*p* = 0.034) and marked diffuse alveolar edema (*p* = 0.034), which support its pivotal role in the molecular mechanisms associated with hypothermia pulmonary edema.

In addition, the overexpression of matrix metalloproteinase 9 (MMP-9), which participates in the degradation of the extracellular matrix and basement membranes, demonstrates its involvement in the imbalance of the fluid flow in the pulmonary interstitium, being associated with disruption of the activity of endothelial AQPs (proteins involved in the regulation of transmembrane water transport) [17]. AQPs are expressed by many cells, including renal tubular epithelial cells and pulmonary capillary alveolar endothelial cells [16,38]. Among these, AQP1 and AQP5 are the most important pulmonary aquaporins, and their overexpression is proven to be associated with interstitial edema in severe hypothermia [17]. AQP5 is intensely expressed by type I pneumocytes, cells that line approximately 90% of the alveolar surface, while AQP1 is expressed by endothelial cells of the alveolar–capillary bed. AQP1 facilitates the development of hydrostatic pulmonary edema but has no involvement in the active reabsorption of intra-alveolar fluids [38], and thus AQP1 gene deletion is associated with a 90% reduction in endothelial permeability and secondary pulmonary interstitial edema development [16]. According to our knowledge, there are only a few studies regarding AQP1 immunoexpression in severe hypothermia, such as the one conducted by Wang et al. on 16 cases of fatal hypothermia, which demonstrated an increased AQP1 endothelium expression in the alveolar septum vessels [17]. These features were partially confirmed by our study when associated with pulmonary emphysema as a possible pre-existing condition, which may have increased the extent and severity of the lesions caused by hypothermia.

Hypothermia is also associated with the disruption of the mucociliary defense and a reduced cough reflex that increases the risk of bronchopneumonia [4,39]. Acute pulmonary infectious complications were also identified in our study group, which was noted in 16 cases. Their occurrence may have also been facilitated by pre-existing lung diseases, such as chronic fibroinflammatory lesions, tuberculosis, or cancer, in our study group.

Our study had some limitations related to the difficulty in discrimination between pre-existing cerebral and pulmonary diseases and their variable associations, along with complications of low-temperature exposure and variable periods of time between the cold exposure and the time of death. Regarding the interpretation of the results, the comparison with normal thermal status could not be achieved, considering the high variability of circumstances of death and related pathologies, with possible brain S100β and Hsp70 expression associated with agonal cerebral edema and neuronal ischemia and lung ICAM-1 and AQP1 expression associated with pulmonary edema related to alveolar and endothelial lesions. However, data interpretation was accomplished following the analysis of patients’ history, with the exclusion of cerebral degenerative diseases or stroke, in the appropriate death circumstances, by the corroboration of available findings. Additionally, due to the numerous variables analyzed, the correlation tests between postmortem immunohistochemical markers’ expression and histopathological characteristics were laborious. Last but not least, the forensic pathological and postmortem immunohistochemical methods were challenging due to the cold deterioration of the tissues added to autolysis and putrefaction.

Moreover, the present study provides supplementary morphological data to the findings already reported regarding cardiomyocyte degenerative changes, kidney Armanni–Ebstein lesions, and tubular necrosis [3], along with pulmonary edema and focal neural ischemia, which may support the diagnosis of hypothermia-related death in suspected cases. Weak sirtuin 1 (SIRT1) myocardium expression, with its loss in contraction band areas and variable ubiquitin (Ub) immunoexpression in renal parenchyma [3], along with S100β and HpS40 strong expression in glial cells and neurons and ICAM-1 and AQP-1 overexpression in alveolar walls are the most common findings in cases of hypothermia fatalities according to our results.

Our findings are in agreement with the current concept that, although none of the forensic pathological features are specific, a hypothermia diagnosis may be achieved by the corroboration of gross, microscopic, and postmortem immunohistochemical characteristics.

In addition, our results support the complexity of molecular mechanisms associated with tissue changes as a result of cold-stress exposure and may add useful data for the development of new diagnosis tools in hypothermia patients.

## 5. Conclusions

Our results provide useful data regarding the possible molecular pathogenic pathways associated with cerebral and pulmonary cellular reactivity in hypothermia victims, based on a morphological analysis applied to a relatively large number of autopsies. Alveolar edema, with intra-alveolar and/or pleural hemorrhage, and alveolar septa ICAM-1 and AQP-1 positive expression, along with subarachnoid hemorrhage, intraparenchymal and perivascular hemorrhages, diffuse perineuronal and perivascular edema, gliosis, mononuclear cell infiltration, focal neuronal ischemia, lacunar infarction, S100β immunopositive expression of glial cells and moderate neurons’ Hsp70 immunopositivity were the most significant microscopic lesions registered in hypothermia-related deaths.

The extension of the studies related to hypothermia would result in an important breakthrough in understanding the fatal respiratory consequences of prolonged exposure to cold, along with the identification of the limitations and the potential use of induced hypothermia in cerebral protection. Our study findings may open new directions of development of a diagnostic algorithm for hypothermia-related fatalities and hypothermia patients’ management, identifying some pathogenic mechanisms that may be specifically addressed by targeted therapy.

## Figures and Tables

**Table 1:** The cerebral gross features in hypothermia-related victims (*n* = 107).

Cerebral Gross Findings		No. of Cases *n* (%); (*n*F; *n*M)
Congestion		74 (69.15%); (16 F; 58 M)
Edema	moderate	94 (87.85%); (22 F; 72 M)
	marked	11 (10.28%); (4 F; 7 M)
Subarachnoid hemorrhage		6 (5.6%); (1 F; 5 M)
Cerebral hemorrhage		1 (0.93%) (0 F; 1 M)
Brain softening		5 (4.67%); (0 F; 5 M)
Cerebral atherosclerosis		17 (15.88%); (5 F; 12 M)
Scalp hematomas		16 (14.95%); (6 F; 10 M)

F—female; M—male; *n*—number of cases.

**Table 2:** Univariate analysis of S100β and Hsp70 postmortem immunoexpression in cerebral samples vs. forensic pathological features in the study group (*n* = 107).

Histopathological Features		S100β		*r*	*p*-Value	Hsp 70		*r*	*p*-Value
		Negative	Positive			Negative	Positive		
Diffuse perineuronal and perivascular edema	Absent	2	0	0.34	0.019	2	0	0.22	0.077
	Present	13	92			28	77		
Arteriolosclerosis	Absent	3	90	−0.80	<0.001	16	77	−0.62	<0.001
	Present	12	2			14	0		
Gliosis and mononuclear cell infiltration	Absent	11	91	−0.42	0.001	25	77	−0.35	0.001
	Present	4	1			5	0		
Acute brain injuries (contusions)	Absent	14	90	−0.09	0.367	27	77	−0.27	0.020
	Present	1	2			3	0		
Meningeal fibrosis	Absent	14	92	−0.24	0.140	29	77	−0.15	0.280
	Present	1	0			1	0		
Focal neuronal ischemia	Absent	13	91	−0.25	0.051	27	77	−0.27	0.020
	Present	2	1			3	0		
Lacunar infarction	Absent	11	92	−0.48	<0.001	26	77	−0.31	0.005
	Present	4	0			4	0		
Subarachnoid hemorrhage	Absent	7	91	−0.65	<0.001	21	77	−0.48	<0.001
	Present	8	1			9	0		
Intraparenchymal and perivascular hemorrhages	Absent	9	85	−0.34	0.003	18	76	−0.53	<0.001
	Present	6	7			12	1		

*p*-value < 0.05; *r*—Pearson’s correlation coefficient.

**Table 3:** The pulmonary gross and microscopic features in hypothermia-related victims (*n* = 106).

Organ	Gross Findings		No. of Cases *n* (%); (*n*F; *n*M)
	Congestion		46 (43.39%); (10 F; 36 M)
	Pulmonary edema	moderate	88 (83.01%) (20 F; 68 M)
		marked	18 (16.98%) (6 F; 12 M)
	Chronic parenchymal and pleural inflammation and fibrosis		39 (36.79%); (11 F; 28 M)
	Purulent inflammation		17 (16.03%); (3 F; 14 M)
	Lung hemorrhages		10 (9.43%); (2 F; 8 M)
	Pleural petechiae		8 (7.54%); (1 F; 7 M)
	Bullous emphysema		3 (2.8%); (1 F; 2 M)

F—female; M—male; *n*—number of cases.

**Table 4:** Univariate analysis of lung ICAM-1 and AQP1 postmortem immunoexpression in lung samples vs. forensic pathological features in the study group (*n* = 106).

Histopathological Features		ICAM-1		*r*	*p*-Value	AQP1		*r*	*p*-Value
		Negative	Positive			Negative	Positive		
Hemorrhagic alveolar edema	Absent	9	9	0.20	0.034	2	16	0.13	0.199
	Present	22	66			3	85		
Marked diffuse pulmonary alveolar edema	Absent	22	66	−0.20	0.034	5	83	0.10	0.586
	Present	9	9			0	18		
Pulmonary emphysema	Absent	24	74	−0.36	0.001	3	95	−0.27	0.045
	Present	7	1			2	6		
Intra-alveolar and/or pleural hemorrhages	Absent	25	64	−0.05	0.569	5	84	0.09	1.000
	Present	6	11			0	17		
Bronchopneumonia	Absent	24	68	−0.17	0.111	4	88	−0.04	0.515
	Present	7	7			1	13		
Bronchopneumonia with abscess formation	Absent	29	75	−0.21	0.084	5	99	0.03	1.000
	Present	2	0			0	2		
Acute respiratory distress syndrome (ARDS)	Absent	30	75	−0.15	0.292	5	100	0.02	1.000
	Present	1	0			0	1		
Pleuritis	Absent	19	75	−0.55	<0.001	4	90	−0.06	0.458
	Present	12	0			1	11		
Pleural adhesions	Absent	21	75	−0.50	<0.001	3	93	−0.23	0.069
	Present	10	0			2	8		
Pulmonary tuberculosis	Absent	30	74	−0.06	0.501	5	99	0.03	1.000
	Present	1	1			0	2		
Chronic fibroinflammatory lesions	Absent	28	74	−0.19	0.074	5	97	0.04	1.000
	Present	3	1			0	4		
Squamous cell carcinoma	Absent	30	75	−0.15	0.292	5	100	0.02	1.000
	Present	1	0			0	1		
Intra-alveolar foreign bodies	Absent	29	75	−0.21	0.084	5	99	0.03	1.000
	Present	2	0			0	2		

*p*-value < 0.05; *r*—Pearson’s correlation coefficient.

## Data Availability

All supporting data are provided in the current manuscript.
